# Stress-driven cardiac calcium mishandling via a kinase-to-kinase crosstalk

**DOI:** 10.1007/s00424-021-02533-2

**Published:** 2021-02-15

**Authors:** Charia McKee, Dan J. Bare, Xun Ai

**Affiliations:** Department of Physiology & Biophysics, Rush University Medical Center, 1750 West Harrison St. 1255 Jelke, Chicago, IL 60612-3825 USA

**Keywords:** c-jun N-terminal kinase, Ca^2+^/calmodulin-dependent protein kinase II, Sarcoplasmic reticulum, Calcium handling, Diastolic calcium leak

## Abstract

Calcium homeostasis in the cardiomyocyte is critical to the regulation of normal cardiac function. Abnormal calcium dynamics such as altered uptake by the sarcoplasmic reticulum (SR) Ca^2+^-ATPase and increased diastolic SR calcium leak are involved in the development of maladaptive cardiac remodeling under pathological conditions. Ca^2+^/calmodulin-dependent protein kinase II-δ (CaMKIIδ) is a well-recognized key molecule in calcium dysregulation in cardiomyocytes. Elevated cellular stress is known as a common feature during pathological remodeling, and c-jun N-terminal kinase (JNK) is an important stress kinase that is activated in response to intrinsic and extrinsic stress stimuli. Our lab recently identified specific actions of JNK isoform 2 (JNK2) in CaMKIIδ expression, activation, and CaMKIIδ-dependent SR Ca^2+^ mishandling in the stressed heart. This review focuses on the current understanding of cardiac SR calcium handling under physiological and pathological conditions as well as the newly identified contribution of the stress kinase JNK2 in CaMKIIδ-dependent SR Ca^2+^ abnormal mishandling. The new findings identifying dual roles of JNK2 in CaMKIIδ expression and activation are also discussed in this review.

## Introduction

In the mammalian heart, calcium (Ca^2+^) is an essential regulator of electrical signals, contractile function, and excitation–contraction coupling (ECC) during each heartbeat and also plays an important role in the cellular signal transduction pathways that control myocyte survival and growth [17, 42, 116]. Under physiological conditions, the heart beats more than two billion times during an average human lifespan to supply blood to the body. With increasing age and abnormal conditions such as heart failure (HF) and excessive alcohol intake, impaired Ca^2+^ homeostasis causes myocardial molecular remodeling including aberrant gene expression, myocyte death, contractile dysfunction, and arrhythmias [2, 111, 150]. Ca^2+^/calmodulin-dependent protein kinase II (CaMKII) is a multifunctional signaling molecule that plays a central role in this impaired Ca^2+^ homeostasis promoting maladaptive cardiac remodeling and arrhythmias [2, 6, 91, 155–157].

Under pathological conditions, intrinsic cellular stresses caused by a number of stimuli including oxidative stress, ischemia, and inflammation are markedly enhanced. These stressors are also well-established risk factors for the development of cardiovascular diseases [12, 14, 62, 64, 103]. The c-jun N-terminal kinase (JNK), a member of the mitogen-activated protein kinase (MAPK) family, is activated in response to various stress challenges [29, 66, 112, 115]. In fact, JNK activation has been observed with aging, excessive binge alcohol intake-triggered “Holiday Heart Syndrome,” and with cardiovascular diseases such as ischemic myocardial infarction (MI) and HF [29, 63, 66, 80, 108, 112, 115, 136, 144]. Our lab recently discovered a novel sub-cellular mechanism describing a pathogenic kinase-to-kinase crosstalk between JNK2 kinase and the “pro-arrhythmic kinase” CaMKIIδ in the governance of intercellular Ca^2+^ signaling and consequently Ca^2+^-mediated arrhythmias.

A JNK kinase was first discovered in the early 1990s by Kyriakis and Avruch and reported as a novel protein called pp54 MAP-2 kinase, which is activated by duel phosphorylation of the amino acid residues of serine-183 and threonine-185 [74, 75]. Later, two isoforms were identified with molecular weights of 46 and 56 kDa and were named JNK1 and JNK2, respectively [58]. It was then revealed that these JNK kinases could be activated by various extracellular stimuli. Because JNK contains the threonine–tyrosine phosphorylation motif (TPY), it was thus characterized as a member of the MAPK family. Next, JNK3 was discovered in 1995 as the third member of this MAPK subfamily and is mainly expressed in neurons [67, 95, 99, 115]. In the heart, JNK1 and JNK2 are the major isoforms, while JNK3 is expressed at a much lower level [73]. In this review, we will discuss the new findings identifying dual roles of JNK2 in CaMKIIδ expression and activation and the current understanding of the crucial role of the stress kinase JNK2 isoform in CaMKIIδ-dependent aberrant sarcoplasmic reticulum (SR) Ca^2+^ handling in stressed hearts.

## Physiological calcium dynamics in the heart

Ca^2+^ is an important cation in the conversion of an electrical signal to mechanical function in the heart from beat to beat [1, 88, 93, 118, 126]. The voltage-gated L-type Ca^2+^ channels (LTCCs) located in the plasma membrane are activated by depolarization of the myocyte membrane, which leads to a small amount of inward Ca^2+^ flux (*I*_Ca_) [44, 50, 81, 117, 123, 130]. This Ca^2+^ entry through LTCCs triggers large quantities of Ca^2+^ to be released from the SR via cardiac ryanodine receptor type-2 (RyR2; also called Ca^2+^-triggered SR Ca^2+^ release channels) [9, 84, 93]. The plasma membrane and SR are coupled to allow this Ca^2+^-induced Ca^2+^ release (CICR), which occurs locally within the clusters of RyR2 channels on the SR membrane that are in close proximity to LTCCs [59, 77]. CICR is further facilitated by dyads, which are the structures consisting of terminal cisternae of SR, composed of RyR2 channels, paired with transverse tubules (T-tubules), and LTCCs [139]. When an action potential arrives at the T-tubule, Ca^2+^ influx via LTCCs activates RyR2 channels on the cytosolic side of the SR allowing the occurrence of CICR, which activates neighboring RyR2 channels within the same dyad, resulting in a rapid increase in cytosolic Ca^2+^ [45, 76]. CICR is also the trigger for Ca^2+^-troponin C binding, leading to myofilament activation and cardiac muscle contraction [86, 89].

During cardiac muscle relaxation, LTCCs close and terminate the influx of Ca^2+^ and cytosolic Ca^2+^ is removed through the sodium–calcium exchanger (NCX) to the extracellular space and pumped back to the SR through the cardiac sarcoplasmic reticulum Ca^2+^-ATPase (SERCA2), while another small portion of Ca^2+^ is taken up by mitochondria via mitochondrial Ca^2+^ uniporters as well as a small Ca^2+^ efflux via the plasma membrane Ca^2+^-ATPase (PMCA) [10, 17, 34, 50, 107]. Normal contraction of the heart requires high Ca^2+^ levels in systole and low levels in diastole [35, 69]. Therefore, SR Ca^2+^ release via RyR2 channels and reuptake via the predominant Ca^2+^ pump SERCA2a isoform (SERCA2a), and to a much lesser extent SERCA2b isoform, critically mediate the cytoplasmic Ca^2+^ concentration, which is essential in cardiac contraction and relaxation of each heartbeat [1, 118].

## Functional impacts of pathological SR Ca^2+^ mishandling

**Fig. 1:**
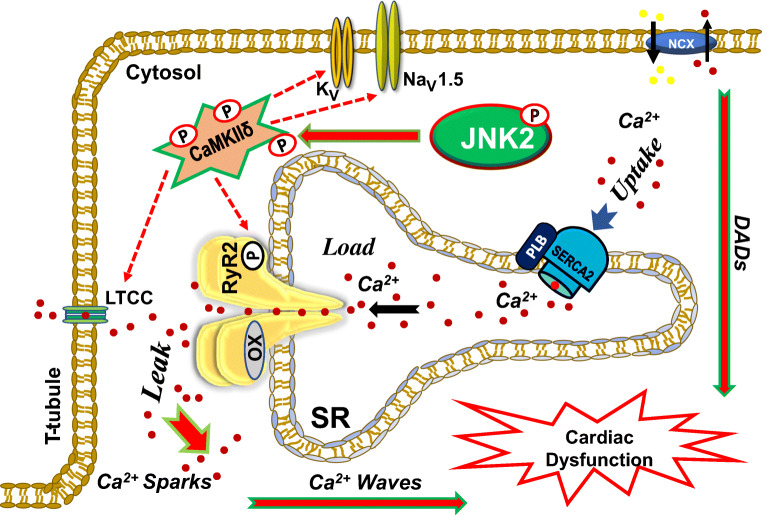
A representation of normal Ca^2^^+^ cycling in cardiomyocytes and stress kinase JNK2-mediated CaMKIIδ-dependent SR Ca^2+^ mishandling and maladaptive cardiac dysfunction. Physiological and pathological regulation of Ca^2+^ cycling in cardiomyocytes and activated CaMKIIδ-dependent dysregulation of RyR2 as well as other ion channels (i.e., Na_V_1.5 and K_V_), which lead to enhanced diastolic SR Ca^2+^ leak, triggered arrhythmic Ca^2+^ activates (sparks and waves), DADs, and cardiac dysfunction. A JNK2-driven CaMKIIδ-dependent diastolic leak–uptake relationship also enhances triggered arrhythmic activities. *JNK2* c-jun N-terminal kinase, *CaMKIIδ* Ca^2+^/calmodulin-kinase type-II delta isoform, *Na*_*V*_*1.5a* voltage-gated sodium channel isoform 1.5-alpha, *LTCC* L-type Ca^2+^ channels, *OX* oxidation, *SR* sarcoplasmic reticulum, *PLB* phospholamban, *SERCA2* SR Ca^2+^-ATPase, *DADs* delayed afterdepolarizations, *P* phosphorylation, *RyR2* ryanodine receptor type-2, *NCX* sodium-calcium exchanger

Given the tightly regulated role of Ca^2+^ in ECC, even a small amount of aberrant Ca^2+^ release resulting from slowly developing pathological changes in the intracellular Ca^2+^ homeostasis can potentially have escalating negative consequences for the myocyte and ultimately the whole heart. Under pathological conditions including HF, ischemia–reperfusion (IR) injury, post-MI, atrial fibrillation (AF), and ventricular arrhythmias, abnormal SR Ca^2+^ dynamics result in electrical and mechanical dysfunctions and myocardial maladaptive function (Fig. 1) [2, 18, 21, 22, 30, 68, 82, 145, 147].

For instance, HF is a common disorder in which the cardiac output does not meet the needs of the body resulting from dysfunctional contractility, impaired electrical conduction, and abnormal energy metabolism [22, 30]. During the SR Ca^2+^ cycling, decreased SR Ca^2+^ refill via reduced Ca^2+^ uptake by SERCA2a leads to a reduced Ca^2+^ transient amplitude and consequently decreased cardiac contractility as seen in the failing heart [57, 83, 125, 142]. In the diastolic phase, SR Ca^2+^ release normally shuts off almost completely (∼99%). However, increased diastolic RyR2 channel activity could be responsible for increased diastolic SR Ca^2+^ leak and reduced systolic fractional Ca^2+^ release for a given L-type voltage-gated Ca^2+^ current (*I*_ca_) as the release trigger [11, 19, 121]. The increased diastolic SR Ca^2+^ leakage along with an impaired SR Ca^2+^ uptake in HF slows down the intracellular Ca^2+^ decline and then elevates the amount of diastolic intracellular Ca^2+^ concentration, which leads to increased sodium (Na^+^) influx via NCX for removing the elevated intracellular Ca^2+^ outside of the cell membrane. As a result, increased diastolic SR Ca^2+^ leak promotes aberrant Ca^2+^ events (Ca^2+^ sparks and waves) and the inward NCX current produces abnormal triggered activities such as delayed after-depolarizations (DADs) and initiates atrial arrhythmias such as atrial fibrillation (AF, the most common cardiac arrhythmia) and ventricular arrhythmias including ventricular tachycardia and ventricular fibrillation (a fatal type of cardiac arrhythmia) [9, 17, 19, 31, 81]. This abnormal SR Ca^2+^ handling also occurs in AF pathogenesis as discussed in detail below.

## Ca^2+^/calmodulin-dependent protein kinase II in the pathological SR Ca^2+^ mishandling

One of the hallmarks of a diseased heart is that altered protein phosphorylation critically contributes to ion transporter and channel dysfunctions, which leads to the disruption of SR Ca^2+^ dynamics. CaMKII is a well-recognized pro-arrhythmic kinase, promoting abnormal SR Ca^2+^ dynamics via phosphorylation of Ca^2+^ handling proteins in the heart. There are four highly conserved isoforms of CaMKII (α, β, γ, and δ) widely expressing in the body, while the δ isoform is predominantly expressed in the heart. Extensive studies demonstrate that activated CaMKIIδ is critically involved in phosphorylation of RyR2 at the site of Ser2815, resulting in sensitized RyR2 channels and profoundly increased diastolic SR Ca^2+^ leak that in turn promotes triggered activities and arrhythmia initiation in pathologically altered ventricles in HF [2, 6, 51, 56, 87, 91, 111, 127, 150, 155, 156, 158]. Although protein kinase A (PKA) has also been shown to phosphorylate RyR2 channels, inconsistent findings regarding the arrhythmic effect of PKA hyperphosphorylation of RyR2 at Ser2809 in HF were reported [3, 7, 16, 85, 90, 110, 120, 135, 138, 140, 154], further suggesting the key role of CaMKIIδ in HF-evoked arrhythmias. In recent years, accumulating evidence suggests that CaMKIIδ-dependent RyR2 channel dysfunction also leads to SR Ca^2+^ mishandling and triggered activities (delayed afterdepolarizations (DADs)) in the atria of chronic AF patients and post-operative AF patients [55, 131]. For instance, activated CaMKII was found to increase both arrhythmic Ca^2+^ activities and profibrotic activity-caused structural remodeling in chronic AF patients associated with HF [28, 96]. In post-operative AF patients (with no history of AF prior to the open-chest surgery), activation of the inflammasome signaling protein, NACHT, LRR, and PYD domain containing protein 3 (NLRP3), was found to augment the CaMKIIδ-dependent RyR2-hyperphosphorylation and arrhythmic Ca^2+^ activities [25, 26, 100]. Further, this CaMKIIδ-dependent SR Ca^2+^ mishandling was recently found to underlie AF pathogenesis in rabbit and mouse models of aging and holiday heart syndrome, in a tachy-pacing canine AF model, and in a spontaneous AF CREM mouse model [33, 79, 134, 145]. Similar to the regulatory actions of CaMKIIδ in the RyR2 activity, CaMKIIδ is known to enhance the Ca^2+^ binding affinity of SERCA2a by phosphorylating phospholamban (PLB) at the threonine-17 site (PLB17) to release the inhibitory PLB from SERCA2a, which enhances SERCA2a Ca^2+^ affinity and stimulates SR Ca^2+^ uptake [124]. The inhibitory effect of PLB on the SERCA2a activity contributes to, at least in part, reduced SR Ca^2+^ uptake in failing hearts [27]. In addition to the critical role of activated CaMKIIδ in SR Ca^2+^ handling, CaMKIIδ is also known to regulate other ion channels such as Ca^2+^ [114, 137], Na^+^ [53, 132, 151], and K^+^ [98, 102] channels and NCX [143, 149]. For instance, CaMKIIδ-dependent phosphorylation of cardiac voltage-gated sodium channel isoform 1.5 (Na_V_1.5) enhances a late depolarizing current (*I*_Na. Late_), which leads to prolonged action potentials (APs) and disrupted Ca^2+^ handling and promotes arrhythmogenic DADs [48, 71]. In addition to Na^+^ channels, it has been suggested that CaMKIIδ activation regulates potassium Kv channels and reduces *I*_to_ and *I*_K1_ currents, which prolong APs and increase Ca^2+^-triggered repolarization, and ultimately enhance arrhythmogenicity [78, 133, 141]. Thus, CaMKIIδ is an important arrhythmic kinase playing a crucial role in the cardiac Ca^2+^ homeostasis and ECC in pathologically remodeled hearts (Fig. 1). Because CaMKIIδ is the predominant pro-arrhythmic isoform in the heart, CaMKIIδ inhibition has been considered a potential therapeutic approach to treat heart diseases [4, 5, 43, 72, 113]. Thus, understanding the underlying mechanisms of CaMKIIδ activation is of vital importance.

## A novel finding of stress kinase JNK2-regulated CaMKIIδ activation

**Fig. 2:**
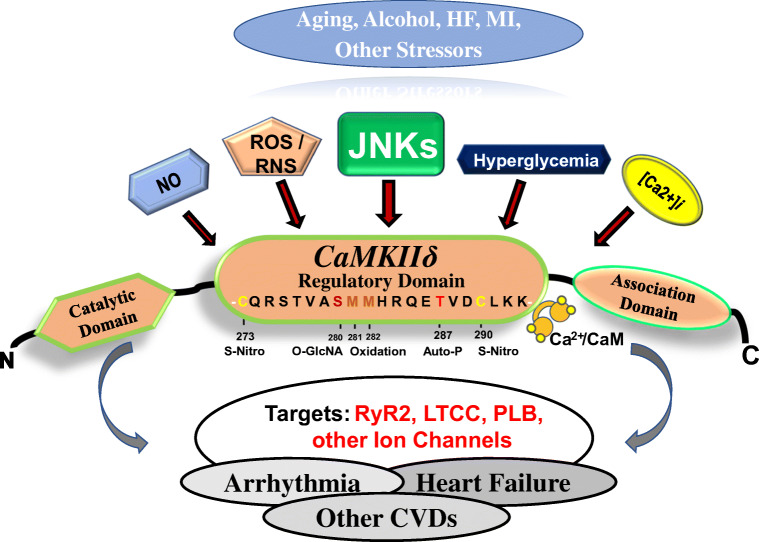
A schematic outline of underlying mechanisms of CaMKIIδ activation and its functional consequences. Stressors (i.e., aging, alcohol, HF, and MI) lead to the activation of signal transduction pathways and the JNK kinase family (JNKs), which enhance CaMKIIδ activity through post-translational modifications including *S*-nitrosylation, *O*-GlcNacylation, oxidation, or direct phosphorylation on the regulatory domain of CaMKIIδ. Activated CaMKIIδ phosphorylates its downstream protein targets (i.e., RyR2, LTCC, and PLB) that are central to calcium homeostasis in the cardiomyocyte and in abnormal calcium dynamics in the development of maladaptive cardiac remodeling under pathological conditions (i.e., arrhythmia and HF). *Auto-P* autophosphorylation, *Ca*^*2+*^/*CaM* calmodulin, *CVDs* cardiovascular diseases, *HF* heart failure, *LTCC* L-type Ca^2+^ channels, *MI* myocardial infarction, *NO* nitric oxide, *O-GlcNA* O-linked-N-acetylglucosaminylation (O-GlcNAcylation), *PLB* phospholamban, *RNS* reactive nitrogen species, *ROS* reactive oxygen species, *RyR2* ryanodine receptor type-2, *S-Nitro* s-nitrosylation.

In the past decades, significant progress has been made regarding the underlying mechanisms of CaMKIIδ activation under pathological conditions [39–41, 52, 155]. CaMKIIδ is a serine/threonine kinase with an increased activity when the site of threonine-287 is phosphorylated that leads to increased binding of its regulatory region to Ca^2+^/calmodulin, exposing CaMKIIδ to the kinase substrate and ATP binding sites and allowing phosphorylation of the target proteins by CaMKIIδ. Several elegant studies [39–41, 52] revealed different underlying mechanisms of post-translational modification including oxidization at the Met281/Met282 sites, *S*-nitrosylation on Cys290, and *O*-GlcNAcylation on Ser279, which all can lead to sustained activation of CaMKIIδ under various pathological conditions (Fig. 2). On the other hand, protein phosphatases such as PP1 are also important in maintaining the activation status of CaMKIIδ in HF and AF [23, 36, 60, 101, 152]. In patients with myocardial infarction and angina, increased protein phosphatase 1 (PP1) and reduced endogenous PP1 inhibitory protein inhibitor-1 (I-1) were associated with reduced CaMKII activity via dephosphorylation of the autophosphorylation site Thr287 of CaMKIIδ, while I-1 KO mice also showed reduced CaMKIIδ activity in the heart [101, 152]. In chronic AF, hyperphosphorylated I-1 suppresses the PP1 activity to consequently sustain the activation of CaMKIIδ [36, 54, 92]. However, reduced I-1 was also found in the failing heart, but it was associated with increased CaMKII activity due to the exchange protein activated by cAMP (EPAC)-augmented CaMKII activation [101]. Moreover, activation of CaMKIIδ could be sustained by reactive oxygen species (ROS) via inactivating phosphatases to reduce the protein phosphatase-regulated dephosphorylation of CaMKIIδ [5, 101]. Clearly, this kinase–phosphatase relationship is complex in the diseased heart and further investigation is needed. Nevertheless, all of the current findings emphasize the clinical significance of exploring effective approaches to inhibit CaMKIIδ activity as potential therapeutic strategies to prevent and/or treat cardiac diseases and arrhythmias.

Recently, our lab reported for the first time that the stress–response kinase JNK2 drives a kinase-to-kinase crosstalk as a previously unrecognized molecular mechanism of CaMKIIδ activation in both aging and binge alcohol-exposed holiday heart syndrome human and animal models [145, 147]. Notably, elevated cellular stress is a common feature of the heart under extrinsic stimuli or during pathological remodeling. JNK is a well-characterized kinase that is activated in response to intrinsic and extrinsic stress stimuli, and then modulates cellular functions including Ca^2+^ mishandling, cell death, and survival [29, 67]. JNK1 activation has been observed in various cardiovascular diseases including IR injury, MI, and HF, which frequently occur together in the aging population [15, 49, 66, 112, 115, 144]. However, the function of JNK2 in the heart has received less attention. Although not all hearts will experience a particular stress, all hearts will inevitably age. The aged heart is also known to be more susceptible to the stresses it may encounter [64]. We found that JNK2, but not JNK1, is significantly activated in both aged human and animal atria, while the levels of total JNK1 and JNK2 proteins were unchanged [144, 145]. Moreover, we found that activated atrial JNK2 is a consistent feature of aged atrium among different species (humans, rabbits, and mice) [144–147]. Furthermore, our recent unpublished data indicates that JNK2 also exhibits elevated activity in aged ventricles. Functionally, we revealed that this age-induced JNK2 activation directly phosphorylates CaMKIIδ to enhance its kinase activity and drive pathology [25, 113]. Intriguingly, we also discovered that JNK2 and CaMKIIδ proteins are tethered with each other and JNK2 increases phosphorylation of CaMKIIδ at the autophosphorylation site Thr287. Since protein phosphatase PP1 is also known to target this Thr287 site to dephosphorylate CaMKII [101, 129, 152], it could be a counterpoint to JNK2 in sustaining the phosphorylation status of CaMKII under stressed conditions. It is clear that this is worthy of further investigation. Accumulating evidence suggests that activation of the stress–response kinase JNK2 represents a common feature in many organs with either acute or chronic alcohol exposure, which contributes to alcohol-caused cell death and tissue injury [8, 37, 38, 104, 148]. Our lab further detailed a previously unknown link between binge alcohol exposure, JNK2 activation, enhancement of CaMKIIδ activity, and atrial arrhythmogenicity in humans and animal models of “holiday heart syndrome” [145]. Note that alcohol can increase ROS production [105] and elevated ROS does promote CaMKIIδ activation by oxidizing CaMKIIδ’s Met280 and Met281 sites, creating a dynamic methionine oxidation pathway for calcium-independent activation of CaMKIIδ [39]. However, our studies demonstrate that the JNK2-specific regulation of CaMKIIδ activation is independent of either intracellular Ca^2+^ concentration or oxidative stress [145, 147]. Therefore, JNK2 has a specific action in the CaMKIIδ activation in the stressed heart (Fig. 2).

Next, we found that JNK2-specific CaMKIIδ activation results in CaMKIIδ-dependent phosphorylation of RyR2815 and PLB17 in both aged and binge alcohol-exposed hearts and the functional consequence of this JNK2-specific regulation is enhanced arrhythmogenic diastolic SR Ca^2+^ activities and AF pathogenesis. Specifically, JNK2 increases diastolic SR Ca^2+^ leak via CaMKIIδ-dependent phosphorylation of RyR2, which sensitizes RyR2 channels, triggers aberrant Ca^2+^ waves, prolongs the intracellular Ca^2+^ decay time constant, enhances spatiotemporal heterogeneity of Ca^2+^ and electrical impulses, and augments AF susceptibility. Studies have also shown that an altered cellular redox balance towards a more oxidized state can also lead to oxidative modifications of RyR2, which promotes diastolic Ca^2+^ leak, arrhythmogenesis, and contractile dysfunction [13, 19, 20, 122, 160]. While we found JNK2-activated CaMKIIδ is independent of ROS, inhibition of either JNK2 or CaMKIIδ eliminates arrhythmogenic activities including enhanced diastolic leak, aberrant Ca^2+^ waves in myocytes, and enhanced arrhythmic susceptibility in intact heart and live animals. Therefore, our findings demonstrate that JNK2 acts as a key pathological node that transduces different stress stimuli and directly activates CaMKIIδ, which promotes SR Ca^2+^ mishandling in the heart and enhances arrhythmic susceptibility. The inter-relationship between ROS and JNK2 in CaMKIIδ activation remains to be determined. Overall, hyperactivation of CaMKIIδ under a stressed and/or diseased state leads to maladaptive cardiac remodeling including channel dysfunction, impaired Ca^2+^ homeostasis, and contractile dysfunction resulting in deteriorated cardiac function and increased risk of arrhythmias as summarized in Fig. 1. Our recent findings are significant because these JNK2-specific actions on CaMKIIδ activity and SR Ca^2+^ mishandling shed new light on modulating JNK2 as a new strategy to target CaMKIIδ activity for preventing and treating arrhythmias. It is clear that further investigation is needed to understand the potential functional roles of JNK2 on other ion channels and the inter-relationship between JNK2 and other kinases or protein phosphatases.

## Transcriptional regulation of JNK2 in CaMKIIδ expression

**Fig. 3:**
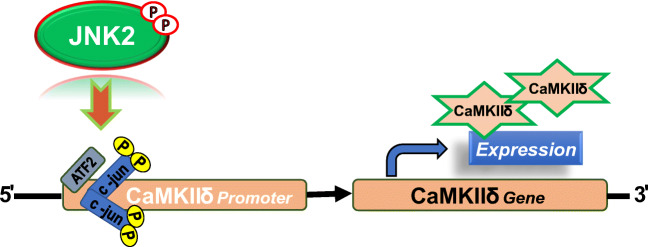
JNK2 kinase is schematically shown as an essential transcriptional regulator of CaMKIIδ expression. The JNK2 downstream transcription factors c-jun and ATF2 both bind to the CaMKIIδ gene promoter, but c-jun is a key transcription factor regulating the basal level expression of CaMKIIδ and a crucial transcriptional enhancer of CaMKIIδ expression in response to certain stress stimuli. *ATF2* activating transcription factor 2

Although CaMKIIδ is essential in regulating a large number of cellular substrates including ion channels, pumps, transporters, and transcription factors [113], how the CaMKIIδ gene and protein expression is controlled remains surprisingly understudied. We recently revealed for the first time that JNK2 plays an essential role in CaMKIIδ expression at the transcriptional level under both physiological and pathological conditions [46] (Fig. 3).

An extensive number of studies demonstrate that JNK1 activation is critically involved in the preservation of cardiac function and in promoting apoptosis after myocardial IR, MI, and HF via the regulation of signaling pathways that modulate gene expression [24, 29, 32, 70, 94, 106, 109, 115, 119, 159]. However, the function of cardiac JNK2, one of the two major cardiac isoforms, has surprisingly received less attention. It is known that JNKs directly regulate these cellular processes via direct phosphorylation of downstream targets and/or indirectly regulate gene expression via downstream transcription factors including c-jun and activating transcription factor 2 (ATF2), forming the activator protein-1 (AP-1) complex [46, 65, 97, 128, 146, 153]. The AP-1 complex is composed of homodimers of c-jun or heterodimers of c-jun/ATF2 or other combinations of transcription factors, which induce target gene expression by binding the AP-1 consensus site(s) in the promoter region of the gene or dissociating from the promoter region to upregulate or suppress the specific gene expression [47]. In our recent studies [46], we discovered that the JNK2 downstream transcription factor c-jun and ATF2 both bind to the CaMKIIδ gene promoter and upregulate CaMKIIδ expression. We further discovered that c-jun is surprisingly a key transcription factor for the basal level expression of CaMKIIδ mRNAs and proteins. This was evidenced by the suppression of CaMKIIδ promoter baseline activity when the c-jun binding consensus sequence was mutated. Moreover, robustly activated JNK2, mimicking a stressed condition, significantly increases the binding of c-jun, but not ATF2, to the CaMKIIδ promoter, while JNK2 inhibition alleviated this enhanced c-jun binding. In addition, the JNK2-specifiic action in c-jun-regulated CaMKIIδ promoter activity was supported by the suppressed CaMKIIδ promoter activity from either JNK2 or c-jun siRNA knockdown. Until very recently, the underlying mechanism of transcriptional regulation of CaMKIIδ gene expression remained completely unknown. Our discovery of the isoform-specific action of JNK2 in CaMKIIδ expression ( 3) provided the first evidence suggesting that JNK2 is not only an essential regulator in CaMKIIδ expression under physiological conditions but also is a crucial transcriptional enhancer in response to certain stress stimuli.

## Conclusions and future directions

Over the years, our understanding of the underlying mechanisms of physiological Ca^2+^ homeostasis in cardiomyocytes and disrupted Ca^2+^ dynamics under pathological conditions has been significantly advanced. However, it is still not completely understood how stress stimuli and stress–response kinase JNKs are involved in aging, alcohol, obesity, and diseased states associated with cardiac Ca^2+^ mishandling and what mechanisms prompt cardiac maladaptive molecular and electrophysiological remodeling and cardiac dysfunction. While numerous studies have significantly advanced our understanding of the key role of hyperactivation of CaMKIIδ in pathological cardiac remodeling and arrhythmia, many questions remain. Examples are the relationship between pathologically hyperactivated CaMKIIδ and stress-activated JNKs, the interaction between JNK2/ CaMKIIδ and their downstream targets (i.e., RyR2, LTCCs, and PLB), and how stress JNK2 signaling and/or CaMKIIδ interact with other pathological signaling pathways during the process of disease development. All of these questions merit further investigation.

Accumulating evidence suggests that suppression of CaMKIIδ function can mitigate arrhythmias and various heart diseases in animal models provoking a great deal of interest in the development of CaMKIIδ inhibitors as possible anti-arrhythmic therapeutic agents [2, 46, 61, 155, 158]. Although a variety of CaMKIIδ inhibitors are currently available for research, their off-target effects hinder their clinical applications [61]. Thus, additional upstream or downstream components of the CaMKIIδ signaling cascades are being considered for new therapeutic approaches. As demonstrated by our recent studies, JNK2 as a key regulator of the pro-arrhythmic CaMKIIδ sheds new light on the possibility of modulating JNK2 activity as an alternative approach to targeting CaMKIIδ activity. This may offer broader clinical applications for treatment of AF, HF, holiday heart syndrome, and potentially other cardiovascular diseases.

## Data Availability

Not applicable.
